# Ketamine-induced apoptosis in the mouse cerebral cortex follows similar characteristic of physiological apoptosis and can be regulated by neuronal activity

**DOI:** 10.1186/s13041-017-0302-2

**Published:** 2017-06-17

**Authors:** Qi Wang, Feng-yan Shen, Rong Zou, Jing-jing Zheng, Xiang Yu, Ying-wei Wang

**Affiliations:** 1 Department of Anesthesiology and Intensive Care Medicine, Xinhua Hospital, College of Medicine, Shanghai Jiaotong University, Shanghai, 200092 China; 2 Department of Anesthesiology, Huashan Hospital, Fudan University, Shanghai, 200040 China; 3 Institute of Neuroscience and State Key Laboratory of Neuroscience, CAS Center for Excellence in Brain Science and Intelligence Technology, Shanghai Institutes for Biological Sciences, Chinese Academy of Sciences, Shanghai, 200031 China; 4 Shanghai Information Center for Life Sciences, Shanghai Institutes for Biological Sciences, Chinese Academy of Sciences, Shanghai, 200031 China

**Keywords:** General anesthesia, Ketamine, Neonates, Neuronal apoptosis

## Abstract

The effects of general anesthetics on inducing neuronal apoptosis during early brain development are well-documented. However, since physiological apoptosis also occurs during this developmental window, it is important to determine whether anesthesia-induced apoptosis targets the same cell population as physiological apoptosis or different cell types altogether. To provide an adequate plane of surgery, ketamine was co-administered with dexmedetomidine. The apoptotic neurons in the mouse primary somatosensory cortex (S1) were quantitated by immunohistochemistry. To explore the effect of neural activity on ketamine-induced apoptosis, the approaches of Designer Receptors Exclusively Activated by Designer Drugs (DREADDs) and an environmental enrichment (EE) were performed. Ketamine-induced apoptosis in S1 is most prominent at postnatal days 5 and 7 (P5 – P7), and becomes insignificant by P12. Physiological and ketamine-induced apoptosis follow similar developmental patterns, mostly comprised of layer V pyramidal neurons at P5 and shifting to mostly layer II to IV GABAergic neurons by P9. Changes in neuronal activity induced by the DREADD system bidirectionally regulated the pattern of ketamine-induced apoptosis, with reduced activity inducing increased apoptosis and shifting the lamination pattern to a more immature form. Importantly, rearing mice in an EE significantly reduced the magnitude of ketamine-induced apoptosis and shifted its developmental pattern to a more mature form. Together, these results demonstrate that lamination pattern and cell-type dependent vulnerability to ketamine-induced apoptosis follow the physiological apoptosis pattern and are age- and activity-dependent. Naturally elevating neuronal activity is a possible method for reducing the adverse effects of general anesthesia.

**Electronic supplementary material:**

The online version of this article (doi:10.1186/s13041-017-0302-2) contains supplementary material, which is available to authorized users.

## Introduction

The potential adverse effects of general anesthetics on the developing brain is an area of major concern for anesthesiologists, surgeons and parents [1–4]. Follow-up studies in patients showed correlations between exposure to general anesthetics at a young age (before the age of 3 or 4) and deficits in learning, motor, language or cognition-related function [1, 5–8]. Consistently, studies in developing rodent and non-human primates have shown that general anesthetics can induce significant neuronal apoptosis, alter synaptic transmission and plasticity, and lead to deficits in learning-related behaviors [7–12]. These results all point to greater vulnerability of the developing brain to the effects of general anesthetics. However, since surgery at young ages is sometime unavoidable, a better understanding of how anesthetics act on the developing brain may help towards reducing their adverse effects.

A well-described effect of general anesthetics is inducing neuronal apoptosis [3, 8, 9, 12]. Apoptosis, a form of programmed cell death, is a process by which cells, upon intrinsic or extrinsic signaling, undergo a characteristic program to actively mediate their own demise [13–15]. In the developing cerebral cortex, physiological apoptosis mostly occurs during the brain growth spurt [16–18]. In rodents, the number of apoptotic neurons in the cerebral cortex peaks during the first postnatal week and becomes essentially undetectable by the end of the third postnatal week [18–20]. Interestingly, peak vulnerability to anesthesia-induced apoptosis also occurs during this time window [3, 7, 17].

Ketamine, a general anesthetic widely used in pediatric surgery, was first shown to induce widespread apoptosis in multiple brain regions of neonatal rats [18]. A variety of general anesthetics have subsequently been shown to have similar effects [3, 8, 9, 12]. The similarity in the time course of physiological and anesthesia-induced apoptosis suggests potential common regulatory mechanisms. To investigate whether this might be the case, we comprehensively examined the effect of ketamine administration on the pattern of neuronal apoptosis in neonatal mice. Moreover, since ketamine mediates its effects primarily by blocking N-Methyl-D-aspartic acid (NMDA) receptors, and the NMDA receptor antagonist MK-801 has also been shown to induce apoptosis [18], we next investigated the effect of neuronal activity on the ketamine-induced apoptosis using the Designer Receptors Exclusively Activated by Designer Drugs (DREADD) system and environmental enrichment (EE).

## Materials and methods

### Animals and experimental procedures

C57BL/6 mice of postnatal ages 5 to 12 (P5– P12) were used. All animal procedures complied with the animal care standards set forth by the US National Institutes of Health and were approved by the Institutional Animal Care and Use Committee of the Institute of Neuroscience, Chinese Academy of Sciences, and Shanghai Jiaotong University. All mice were reared on a 12 h light/12 h dark cycle in temperature- and humidity-controlled rooms. Both male and female mice were used. GAD67-GFP mice were gifts of Prof. Yuchio Yanagawa of Gunma University in Japan [21].

### Anesthesia

Littermate pups were randomly assigned to control or anesthesia groups. Control pups were intraperitoneally injected with phosphate-buffered saline (PBS). Pups in the anesthesia groups were intraperitoneally injected with ketamine (Gutian Pharmaceuticals, Fujian, China), dexmedetomidine (Guorui Pharmaceuticals, Sichuan, China), a selective agonist of α2-adrenergic receptors [22, 23] or ketamine plus dexmedetomidine. The ketamine plus dexmedetomidine combination was used, because when pups were anesthetized with the relatively low dose of 30 mg/kg ketamine, they displayed constant paddling motion with their limbs, for nearly 1 h after ketamine injection. When the ketamine dosage was increased to 90 mg/kg, the paddling motions were significantly reduced, but the pups’ arterial partial pressure of oxygen (PaO_2_) and oxygen saturation (SaO_2_) were significantly lower than normal level, indicating that this dose was too high for maintaining normal oxygenation and respiration (Additional file 2: Table S1). To provide an adequate surgical plane of anesthesia, while maintaining normal oxygenation, we combined ketamine (30 mg/kg) with dexmedetomidine (20 μg/kg), as is commonly used in surgery, especially for children [24–26]. The injection volume was 12 μl/g for all experiments. After saline or drug administration, pups from the control and anesthetized groups were housed in temperature-controlled chambers (each group individually) to maintain their body temperature at 37 °C. In early experiments, an additional control group (PBS#) consisting of pups returned to the dams immediately after PBS injection was also included. No significant differences were observed between PBS and PBS# groups. Unless otherwise stated, 30 mg/kg ketamine, in combination with 20 μg/kg dexmedetomidine was used. For experiments using GAD67-GFP mice, 60 mg/kg ketamine, in combination with 20 μg/kg dexmedetomidine was used, to obtain a higher number of cleaved caspase-3-positive (CC3^+^) cells.

### Immunohistochemistry, image acquisition and analysis

Six hours following saline or anesthesia injections, all pups were deeply anaesthetized with 0.7% sodium pentobarbital and perfused with 0.9% saline, followed by 4% paraformaldehyde (PFA) in PBS. Brains were dissected and post-fixed in 4% PFA/PBS for 4–6 h at 4 °C, and equilibrated in 30% sucrose. Coronal sections (30 μm) containing the primary somatosensory cortex (S1) were cut with a Leica CM1950 cryostat (Wetzlar, Germany), and every 5th section was immunostained. Sections were incubated in blocking solution containing 5% bovine serum albumin (BSA) and 0.5% Triton X-100 for 2 h at 37 °C. Primary rabbit monoclonal antibody against cleaved caspase-3 (CC3, 9661 L, 1:400; Cell Signaling Technology, Beverly, MA, USA) was applied overnight in 0.3% BSA at 4 °C. After washing 3 times in PBS, sections were incubated in Alexa Fluor-conjugated (488 or 568 nm) secondary antibody at 1:500 (Thermo Fisher Scientific, Waltham, MA, USA) for 2 h at 37 °C. Nuclei were labeled with TO-PRO-3 (TOPRO, T3605, 1:10,000; Thermo Fisher Scientific) for 30 min at 37 °C. NeuroTrace 640/660 deep-red fluorescent Nissl staining (N21483, 1:500; Thermo Fisher Scientific) was used to label the cell body of neurons.

For quantitation of the number of CC3^+^cells, images were acquired with a Zeiss Pascal confocal microscope (Jena, Germany) and a 10× Fluor objective (N.A. = 0.5), or a Nikon NiE-A1 plus confocal microscope (Tokyo, Japan) and a 10× Fluor objective (N.A. = 0.45), both with a Z-step of 5 μm. Ten S1-containing sections, covering the entire S1 region, were imaged per pup. To quantitate co-localization between CC3^+^cells and GAD67-GFP, images were acquired with a Nikon NiE-A1 plus confocal microscope with a 40× Plan Fluor oil immersion objective (N.A. = 1.3), at a Z-step of 0.6 μm. Images showing co-localization between CC3^+^ cells and fluorescent markers were acquired with a Nikon NiE-A1 plus confocal microscope and a 20× Fluor objective (N.A. = 0.75), at a Z-step of 2 μm.

Images were analyzed blinded to the experimental condition using ImageProPlus software (Media-Cybernetics, Rockville, MD, USA). Cells were considered to be CC3^+^ if they were above threshold size and displayed detectable cellular outline. TOPRO staining was used to quantitate image area and help define cortical lamination. For analysis of co-localization between CC3 and GAD67-GFP, all layers of Z-stacks were individually examined to ensure that co-localization was bona fide. Approximately 40 CC3^+^ cells were imaged and analyzed per pup. For analysis of the proportion of CC3^+^ cells with Nissl or mCitrine, all images were thresholded before counting, and co-localization was defined as overlap between the thresholded areas. Ten image frames were analyzed per pup in all experiments. Brain slices from control and anesthetized mice from each experiment were co-processed for all immunostaining, imaging and analysis steps.

### Arterial blood gas measurements

Arterial blood gas was measured in P7 pups at 0.5 h or 2.5 h following ketamine anesthesia. Arterial blood was obtained by transcardiac aspiration from the left ventricle using a heparinized 32-gauge hypodermic needle. pH, partial pressure of carbon dioxide (PaCO_2_), PaO_2_, SaO_2_, and HCO_3_^−^ concentration were measured immediately after arterial blood collection, using a portable clinical analyzer (ABL800FLEX, Radiometer, Copenhagen, Denmark).

### In vivo stereotaxic injections and clozapine-N-oxide treatment

P0 pups were anesthetized by cooling on ice. Adeno-associated viruses (AAVs, type 2/8, 2–5 × 10^12^ TU/ml, packaged by Obio Technology, Shanghai, China) were injected bilaterally into S1 at a speed of 0.1 μl/min; 1 μl AAV was injected per hemisphere, as previously described [27]. pAAV-hSyn-HA-hM3D(Gq)-IRES-mCitrine (AAV-hM3Dq-mCitrine; Addgene plasmid 50,463) and pAAV-hSyn-HA-hM4D (Gi)-IRES-mCitrine (AAV-hM4Di-mCitrine; Addgene plasmid 50,464) were gifts of Prof. Bryan Roth, University of North Carolina [28]. Pups were returned to dams after fully awaking from anesthesia. From P5 to P9, littermates expressing hM3Dq-mCitrine or hM4Di-mCitrine were randomly assigned to clozapine-N-oxide (CNO, 1 mg/kg, 12 μl/g) or saline vehicle (12 μl/g) groups and intraperitoneally injected twice per day.

### Environmental enrichment paradigm

The EE paradigm for neonatal mice was as previously described [27, 29, 30]. Briefly, pregnant dams (1 for control; 2 for EE) were randomly assigned to standard control or EE housing at embryonic days 16–20 (E16–20). Control cages are standard mice cages, while EE cages are larger cages containing objects of various shapes and textures, repositioned daily and completely substituted weekly. EE cages had twice as many pups as control cages at all times to ensure increased social interactions.

### Statistical analysis

Statistical analyses were performed using Graph Pad Prism 5 (Graph Pad Software, La Jolla, CA, USA). Unpaired *t*-test (comparing two conditions), one-way ANOVA (comparing three or more conditions) followed by Dunnett’s (all conditions compared to control) or Turkey’s (when comparisons between conditions were necessary) multiple comparisons test, and two-way ANOVA (for comparing two independent variables) followed by Bonferroni’s multiple comparisons test were used for assessing statistical significance, as specified in figure legends. Data are shown as mean ± SEM. All conditions statistically different from control are indicated. ^*^ *P* < 0.05; ^**^ *P* < 0.01; ^***^ *P* < 0.001.

## Results

### The magnitude and laminar pattern of ketamine-induced neuronal apoptosis in S1 are age-dependent

Previous studies on the time course of physiological and ketamine-induced apoptosis in the rodent cerebral cortex suggested that it mostly occurred during the first few weeks of postnatal development [18]. To define this window more precisely, mice pups of ages P5, P7, P9 and P12 were randomly assigned to receive a single i.p. injection of PBS, 30 mg/kg of ketamine (Keta), 20 μg/kg of dexmedetomidine (Dex) or the two drugs combined (Keta + Dex).

**Fig. 1:**
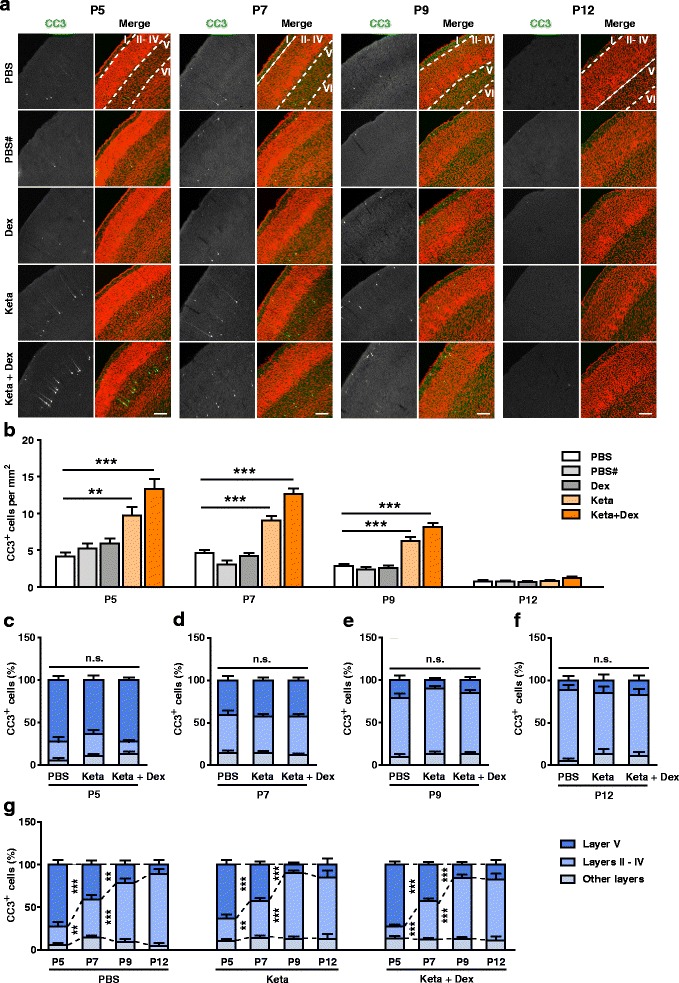
During development, ketamine-induced apoptosis follows the lamination pattern of physiological apoptosis. **a** Representative confocal images of coronal S1 sections immunolabelled for CC3 (green), and co-stained for nuclei with TOPRO (red) to visualize cortical lamination. Age and treatment conditions as indicated. Cortical layers are indicated by Roman numerals, and borders between cortical layers marked using dotted lines. Scale bar is 200 μm. **b** Quantitation of the number of CC3^+^ cells per mm^2^ S1, age and treatment conditions as indicated. PBS and PBS# conditions respectively represent pups injected with PBS and kept away from or together with their mothers during the experiment. Dex, Keta and Keta + Dex respectively represent pups injected with dexmedetomidine, ketamine or ketamine plus dexmedetomidine. All conditions compared to PBS condition of the same age, ^**^ *P* < 0.01, ^***^ *P* < 0.001, using one-way ANOVA followed by Dunnett’s multiple comparisons test. (**c-f**) Distribution of the proportion of CC3^+^ cells in different cortical layers at P5 (**c**), P7 (**d**), P9 (**e**) and P12 (**f**). In all age groups, the percentage of CC3^+^ neurons in layers II – IV, layer V, or other layers (layer I and layer VI) in the Keta group or Keta + Dex group were not significantly different from that of the PBS control group. *P* > 0.05, using two-way ANOVA followed by Bonferroni post hoc tests. (**g**) Replot of the data presented in (**c**-**f**) for the PBS, Keta, and Keta + Dex groups, showing developmental changes in apoptosis pattern. ^**^ *P* < 0.01, ^***^ *P* < 0.001, using two-way ANOVA followed by Bonferroni post hoc tests. 3–5 mice were used per condition, 10 brain slices from each mouse were quantitated. Data are shown as the mean ± SEM

Six hours after injections, pups were perfused and the extent of apoptosis was quantified. Caspase-3 is a key mediator of apoptosis, whose cleavage and activation is often defined as the point of no return for a cell’s commitment to the cell death program [31–33]. Thus, CC3 is commonly used as a marker for apoptotic cells [34]. We quantified the number of CC3^+^ cells in the mouse S1, a brain region that undergoes rapid activity-dependent development during the first two postnatal weeks [35, 36]. In control PBS and PBS# groups, the number of CC3^+^ cells was highest during P5 – P9, and was reduced to negligible levels by P12 (Fig. 1a, b). The PBS and PBS# groups differed in whether pups stayed away from or together with their mothers during the post-injection window (see Methods for details). Since no significant differences were detected between these groups, and anesthetized mice need to be kept away from their mothers before fully awakening, the PBS control was used in all subsequent experiments. Ketamine injection significantly increased the number of CC3^+^ cells in S1 from P5 to P9, as compared to control PBS injected pups (P5, PBS: 4.17 ± 0.48 /mm^2^, Keta: 9.75 ± 1.13 /mm^2^, *P* < 0.01; P7, PBS: 4.62 ± 0.43 /mm^2^, Keta: 9.08 ± 0.55 /mm^2^, *P* < 0.001; P9, PBS: 2.87 ± 0.28 /mm^2^, Keta: 6.27 ± 0.52/mm^2^, *P* < 0.001; Fig. 1a, b). The extent of apoptosis was further increased in pups injected with ketamine plus dexmedetomidine (P5, PBS: 4.17 ± 0.48 /mm^2^, Keta + Dex: 13.28 ± 1.38 /mm^2^, *P* < 0.001; P7, PBS: 4.62 ± 0.43/mm^2^, Keta + Dex: 12.65 ± 0.77/mm^2^, *P* < 0.001; P9, PBS: 2.87 ± 0.28/mm^2^, Keta + Dex: 8.14 ± 0.55/mm^2^, *P* < 0.001; Fig. 1a, b), likely due to increased depth of anesthesia. Injection of dexmedetomidine alone did not increase the number of CC3^+^ cells, as compared to PBS injection alone (Fig. 1a, b). By P12, the number of CC3^+^ cells was low in all conditions and was not significantly different between conditions (Fig. 1a, b). Thus in all subsequent experiments, we focused on the P5 to P9 age range.

To investigate if the laminar pattern of apoptotic cells changed during development, we quantitated the proportion of CC3^+^ cells in different cortical layers. The results showed that at P5, CC3^+^ cells were mostly localized to layer V (PBS: 72.50 ± 5.28%, Keta: 63.49 ± 5.57%, Keta + Dex: 72.56 ± 3.48%, *P* > 0.05), while at P9 and P12, CC3^+^ cells mostly localized to layers II – IV (P9, PBS: 69.22 ± 5.31%, Keta: 76.95 ± 3.15%, Keta + Dex: 71.55 ± 3.77%, *P* > 0.05; Fig. 1a–f). P7 was somewhat in between, with individual pups displaying P5 or P9 lamination patterns or sometimes a mix of the two (Fig. 1a, d).

Across all ages, the PBS control, ketamine and ketamine plus dexmedetomidine groups had similar patterns of CC3^+^ cells across cortical layers, suggesting that ketamine-induced anesthesia did not alter the physiological pattern of apoptosis during development. To better visualize developmental changes in the pattern of apoptosis, we also plotted the proportion of CC3^+^ cells across ages. A significant shift from layer V to layers II - IV CC3^+^ cells was observed for the P5 to P7 transition, and still further for the P7 to P9 transition, independent of whether the mice were anesthetized (Fig. 1g). For all ages and conditions, a small proportion of CC3^+^ cells was observed in layers I and VI, but the percentage did not change with age or treatment condition (Fig. 1).

**Fig. 2:**
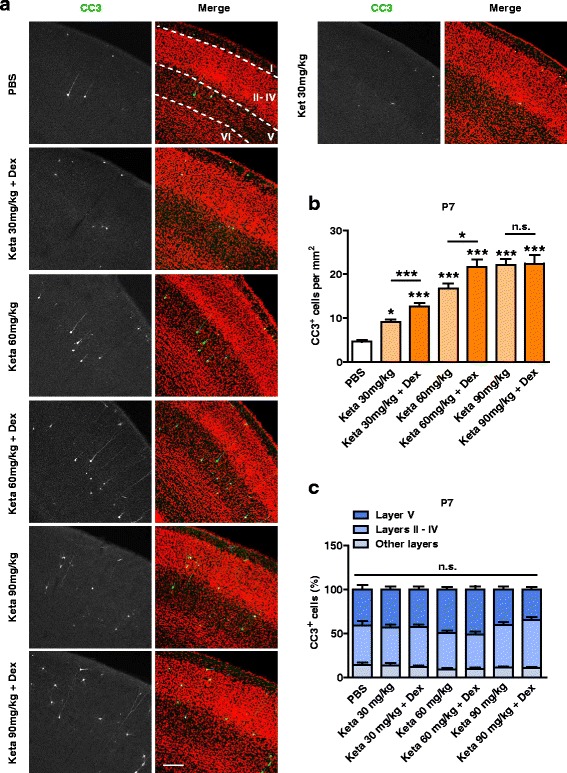
The effect of ketamine on apoptosis is dose-dependent. **a** Representative confocal images of coronal S1 sections immunolabelled for CC3 (green), and co-stained for nuclei with TOPRO (red) to visualize cortical lamination. Treatment conditions as indicated. Cortical layers are indicated by Roman numerals, and borders between cortical layers marked using dotted lines. Scale bar is 200 μm. **b** Quantitation of the number of CC3^+^ cells per mm^2^ S1, treatment conditions as indicated. The number of CC3^+^ cells increased with ketamine dosage. ^*^ *P* < 0.05, ^***^ *P* < 0.001, n.s., not significant, using one-way ANOVA followed by Turkey’s multiple comparison test. **c** The distribution pattern of CC3^+^ cells in different cortical layers was not significantly affected by ketamine dosage. n.s., not significant, using two-way ANOVA followed by Bonferroni post hoc test. The results for PBS, Keta 30 mg/kg and Keta 30 mg/kg + Dex groups are the same as those presented in Fig. 1b and d. 3–6 mice were used per condition, 10 brain slices from each mouse were quantitated. Data are shown as the mean ± SEM

The use of 30 mg/kg ketamine plus 20 μg/kg dexmedetomidine was initially selected, because in our hands, it is the minimal dose required for fully anesthetizing young mice. To further characterize the effect of ketamine at apoptosis, P7 pups were administered with ketamine at the doses of 30 mg/kg, 60 mg/kg or 90 mg/kg, with or without dexmedetomidine (20 μg/kg). The number of CC3^+^ cells increased with ketamine dosage, plateauing out by 90 mg/kg ketamine. Co-administration of dexmedetomidine abolished paddling movements in pups, and when combined with the lower doses of 30 and 60 mg/kg ketamine, also increased the number of CC3^+^ cells as compared with ketamine alone, consistent with it increasing the depth of anesthesia (Fig. 2a, b; please also see Discussion). Importantly, the laminar pattern of ketamine-induced neuronal apoptosis was not affected by drug dosage (Fig. 2c).

To ensure that the level of ketamine and dexmedetomidine used in our experiments did not induce hypoxia or hypercapnia, we measured arterial blood gas parameters of P7 ketamine treated mice at 0.5 h or 2.5 h post-injections. We found that injections of 30 or 60 mg/kg ketamine, alone or in combination with dexmedetomidine, did not significantly influence arterial blood levels of PaCO_2_, PaO_2_, SaO_2_, pH or HCO_3_^−^ (Additional file 2: Table S1). When 90 mg/kg ketamine was used in combination with dexmedetomidine, both pH and SaO_2_ were significantly reduced, suggesting that pups could not maintain normal respiration and oxygenation under these conditions (Additional file 2: Table S1). In all subsequent experiments, 30 mg/kg or 60 mg/kg ketamine, in combination with dexmedetomidine, was used.

### Changes in the vulnerability of GABAergic neurons to apoptosis during development

**Fig. 3:**
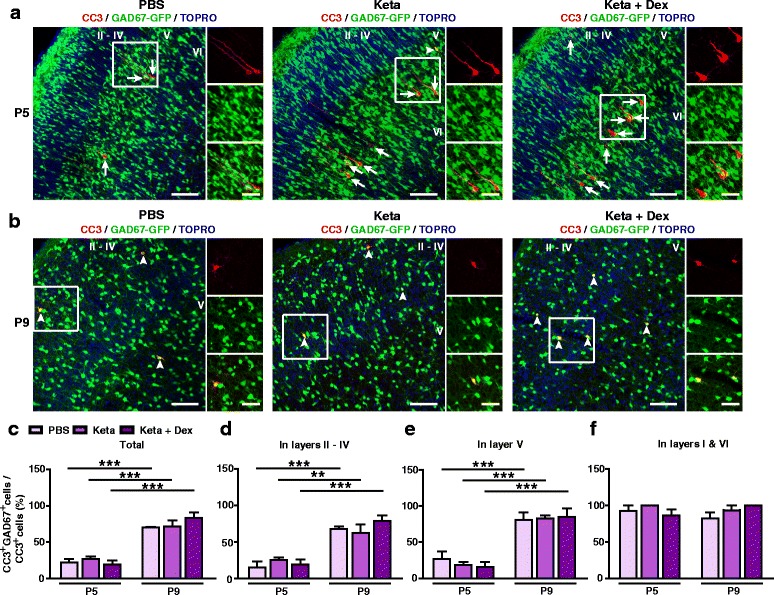
Susceptibility of GABAergic neurons to ketamine-induced apoptosis is age-dependent. **a**, **b** Representative confocal images of S1 sections labelled with CC3 (red), GAD67-GFP (green) and TOPRO (blue) at P5 (**a**) and P9 (**b**). Cortical layers are indicated by Roman numerals. Conditions as indicated. CC3^+^ cells co-localizing with GAD67-GFP are indicated by white arrowheads, while those not co-localizing are indicated by white arrows; scale bar is 100 μm. Zoomed images of boxed regions are presented to the right of each panel; scale bar is 50 μm. (**c**-**f**) Proportion of CC3^+^ cells co-labelling with GAD67-GFP in all cortical layers (**c**), layers II – IV (**d**), layer V (**e**) and layers I and VI (**f**). 4–6 mice were used per condition. ^**^ *P* < 0.01, ^***^ *P* < 0.001, using two-way ANOVA followed by Bonferroni post hoc tests. Data are shown as the mean ± SEM

Having shown that the laminar pattern of physiological and ketamine-induced apoptosis changed during development, we next asked if the cell-type vulnerability to apoptosis was also developmentally regulated. We noticed that at P5, most CC3^+^ cells in layer V exhibited typical pyramidal neuron morphology, with a triangular cells body and a long apical dendrite (Fig. 3a). By P9, however, mostly CC3^+^ cells were found in layers II and IV and were not pyramidal-like (Fig. 3b). We thus asked if these cells were GABAergic interneurons, and tested our hypothesis using GAD67-GFP transgenic mice [21]. Consistent with our initial observations, at P5, less than one third of apoptotic neurons were GAD67-positive (GAD67^+^) interneurons (PBS: 22.61 ± 4.67%, Keta: 27.09 ± 3.74%, Keta + Dex: 19.69 ± 5.27%; Fig. 3a, c), and no significant differences were observed between PBS, ketamine and ketamine plus dexmedetomidine groups (*P* > 0.05; Fig. 3a, c). By P9, however, the majority of CC3^+^ cells were GAD67-GFP^+^ interneurons (PBS: 70.33 ± 0.88%, Keta: 71.31 ± 8.75%, Keta + Dex: 83.24 ± 7.36%; Fig. 3b, c), also with no significant differences between treatment groups (*P* > 0.05). When neurons were subdivided into layers II – IV and layer V, these differences were maintained (Fig. 3d, e). In layers I and VI, GAD67-GFP^+^ cells were almost exclusively GAD67-GFP^+^ at both P5 and P9, but given the small number of CC3^+^ cells in these layers, they did not contribute significantly to the total (Fig. 3f). Not only the proportion of CC3^+^GAD67^+^ neurons increased from P5 to P9 (Fig. 3a-c), the number of apoptotic GABAergic neurons also increased (P5 vs P9: PBS, 0.94 ± 0.11/mm^2^ vs 2.02 ± 0.20/mm^2^, *P* < 0.05; Keta, 3.69 ± 0.32/mm^2^ vs 7.11 ± 0.53 /mm^2^, *P* < 0.01; Keta + Dex, 4.27 ± 0.33/mm^2^ vs 8.35 ± 0.75/mm^2^, *P* < 0.01; *N* = 4–6 mice per condition; two-way ANOVA followed by Bonferroni post hoc tests).

Together, these results demonstrate that the cell-type specificity of physiological and ketamine-induced apoptosis in the S1 was age-dependent, with the majority of apoptotic neurons being pyramidal neurons at P5, and being GABAergic interneurons at P9. Ketamine treatment did not significantly affect the developmental switch in the vulnerability to apoptosis of different cell types.

### Neuronal activity bidirectionally regulates ketamine-induced neuronal apoptosis

**Fig. 4:**
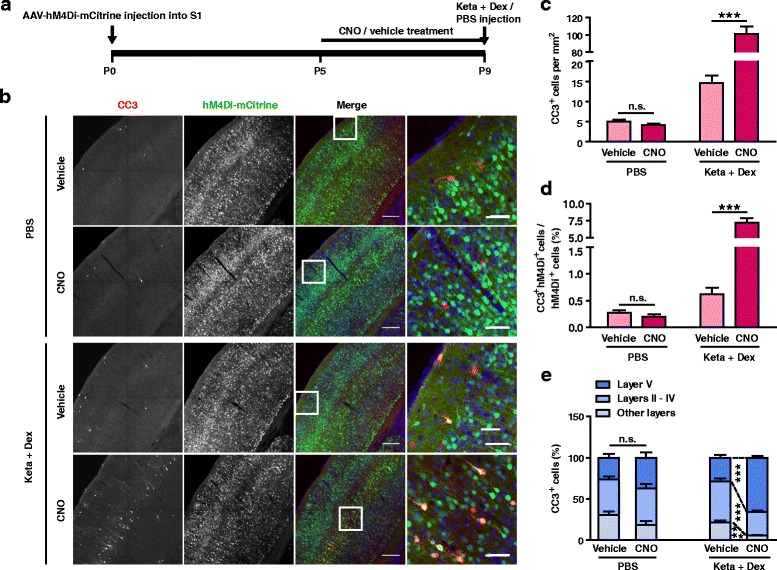
Reducing neuronal activity using hM4Di/CNO significantly enhanced ketamine-induced neuronal apoptosis. **a** Schematic of the experimental procedure. **b** Representative confocal images of S1 sections from P9 mice labelled for CC3 (red), hM4Di-mCitrine (green), and Nissl (blue), conditions as indicated; scale bar is 200 μm. Zoomed images of boxed regions are presented to the right of each panel; scale bar is 50 μm. **c** Quantitation of the number of CC3^+^ cell per mm^2^ S1, treatment conditions as indicated. hM4Di/CNO treatment significantly increased the number of CC3^+^ cells in the Keta + Dex group, but not in control PBS group. ^***^ *P* < 0.001, n.s., not significant, using two-way ANOVA followed by Bonferroni post hoc test. **d** Proportion of hM4Di-mCitrine^+^ cells that are CC3^+^, conditions as indicated. hM4Di/CNO treatment significantly increased the percentage of CC3^+^ cells in Keta + Dex group, but not in the control PBS group. ^***^ *P* < 0.001, n.s., not significant, using two-way ANOVA followed by Bonferroni post hoc test. **e** Distribution of CC3^+^ cells in different cortical layers. ^***^ *P* < 0.001, n.s., not significant, using two-way ANOVA followed by Bonferroni post hoc test. 3 mice were used per condition, 10 brain slices from each mouse were quantitated. Data are shown as the mean ± SEM

Previous studies have shown that systemic injection of the NMDA receptor antagonists MK801, phencyclidine (PCP) or carboxypiperazin-4-yl-propyl-1-phosphonic acid (CPP) significantly increased apoptosis in rodent pups [18, 37], suggesting an important role of neuronal activity in regulating apoptosis. Since systemic drug administration can have complex effects on the activity of neural circuits, we examined whether reducing the activity of individual neurons in S1 using the DREADD system [38], affected their chances of undergoing apoptosis. hM4Di is a G-protein-coupled receptor activated exclusively by its synthetic ligand CNO, and functions by activating G protein-coupled inwardly rectifying K^+^ channels to reduce neuronal spiking [39, 40]. AAV-hM4Di-mCitrine were injected into the S1 of P0 mice, followed by i.p. injections of CNO or vehicle from P5 to P9, and the number of CC3^+^ cells was quantitated at P9 (Fig. 4a). AAV-hM4Di-mCitrine expression was detected in approximately 15% of Nissl^+^ cells, and there were no significant differences between treatment conditions (PBS, vehicle: 15.93 ± 1.33%, CNO: 15.73 ± 1.30%; Keta + Dex, vehicle: 12.89 ± 1.01%, CNO: 15.31 ± 1.68%; *P* > 0.05, two-way ANOVA followed by Bonferroni’s multiple comparisons test; Additional file 1: Figure S1A). Similar number of CC3^+^ cells in AAV-hM4Di-mCitrine expressing S1 regions were present in PBS injected mice, whether they were treated with CNO or vehicle (Fig. 4b, c). For mice injected with ketamine plus dexmedetomidine, however, CNO treatment induced a dramatic increase in the number of CC3^+^ cells (vehicle: 14.55 ± 1.86/mm^2^, CNO: 100.84 ± 8.85/mm^2^, *P* < 0.001, Fig. 4b, c). Similar increases were found when cells double labelled with hM4Di-mCitrine^+^ and CC3^+^ were quantitated (vehicle: 0.62 ± 0.12%, CNO: 7.23 ± 0.65%, *P* < 0.001; Fig. 4d). Quantifying the number of CC3^+^ cells that were not hM4Di^+^ (Keta + Dex, vehicle: 11.95 ± 1.38/mm^2^, CNO: 62.55 ± 4.81/mm^2^, *P* < 0.001, unpaired *t*-test), we found that they were also significantly increased, suggesting that hM4Di expression increased the likelihood of neighboring cells undergoing apoptosis, possibly through effects on the local network activity.

Reducing neuronal activity through hM4Di expression also significantly altered the laminar distribution of CC3^+^ cells, reducing the proportion of layer II – IV cells (vehicle: 49.71 ± 3.33%, CNO: 28.66 ± 1.67%, *P* < 0.001; Fig. 4e) and increasing the proportion of layer V cells (vehicle: 28.53 ± 3.19%, CNO: 65.63 ± 1.91%, *P* < 0.001; Fig. 4e). The laminar pattern in the CNO treated, hM4Di^+^ group resembles that at the earlier developmental stage of P5 (Fig. 1a, c). Together these results demonstrate that reducing neuronal activity significantly increased ketamine-induced neuronal apoptosis and shifted the laminar pattern of apoptosis towards a more immature pattern.

**Fig. 5:**
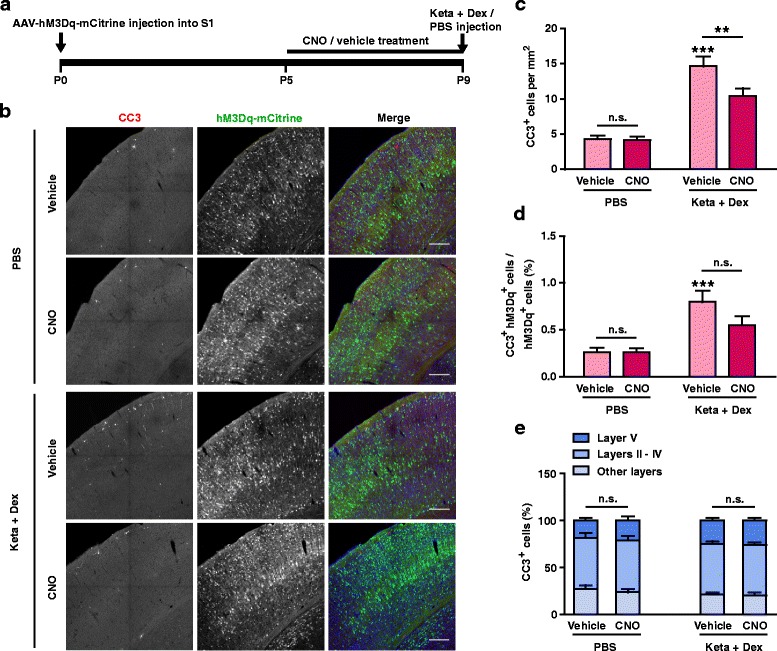
Elevating neuronal activity with hM3Dq/CNO attenuated ketamine-induced apoptosis. **a** Schematic of the experimental procedure. **b** Representative confocal images of S1 sections from P9 mice labelled with CC3 (red), hM3Dq-mCitrine (green), and Nissl (blue). Conditions as indicated. Scale bar is 200 μm. **c** Quantitation of CC3^+^ cell number per mm^2^ S1, treatment conditions as indicated. hM3Dq/CNO treatment significantly reduced the number of CC3^+^ cells in the Keta + Dex group, but not in the PBS group. ^**^ *P* < 0.01, ^***^ *P* < 0.001, n.s., not significant, using two-way ANOVA followed by Bonferroni post hoc test. **d** Proportion of hM3Dq-mCitrine^+^ cells that are CC3^+^, conditions as indicated. ^***^ *P* < 0.001, n.s., not significant, using two-way ANOVA followed by Bonferroni post hoc test. **e** Distribution of CC3^+^ cells in different cortical layers. n.s., not significant, using two-way ANOVA followed by Bonferroni post hoc test. 4 mice were used per condition, 10 brain slices from each mouse were quantitated. Data are shown as the mean ± SEM

An immediately arising question is whether increasing neuronal activity could reduce apoptosis. To examine this, we used another member of the DREADD toolbox, hM3Dq [38, 41, 42], which upon CNO activation, activates the Gq pathway to increase intracellular calcium release and promote neuronal firing [42, 43]. Using a protocol similar to the previous experiment (Fig. 5a), AAV-hM3Dq-mCitrine expression was detected in approximately 10% of Nissl^+^ cells and there were no significant differences between treatment conditions (PBS, vehicle: 8.77 ± 0.65%, CNO: 9.02 ± 0.61%; Keta + Dex, vehicle: 7.80 ± 0.47%, CNO: 9.33 ± 0.57%; *P* > 0.05; two-way ANOVA followed by Bonferroni’s multiple comparisons test; Additional file 1: Figure S1B). AAV-hM3Dq-mCitrine expression also did not significantly affect neuronal apoptosis in control PBS injected mice at P9 (Fig. 5b, c). Upon ketamine plus dexmedetomidine treatment, however, significantly reduced neuronal apoptosis was observed in hM3Dq expressing mice treated with CNO (vehicle: 14.64 ± 1.37/mm^2^, CNO: 10.42 ± 1.10/mm^2^, *P* < 0.01; Fig. 5b, c). Consistently, in the ketamine group, the apoptotic proportion of hM3Dq-expressing neurons also trended towards reduction in the CNO-treated group (Fig. 5b, d). The laminar distribution of apoptotic neurons was not affected (Fig. 5e), consistent with similar laminar patterning of apoptotic cells between P9 and P12 mice (Fig. 1e, f).

### Environmental enrichment reduced ketamine-induced apoptosis in S1

**Fig. 6:**
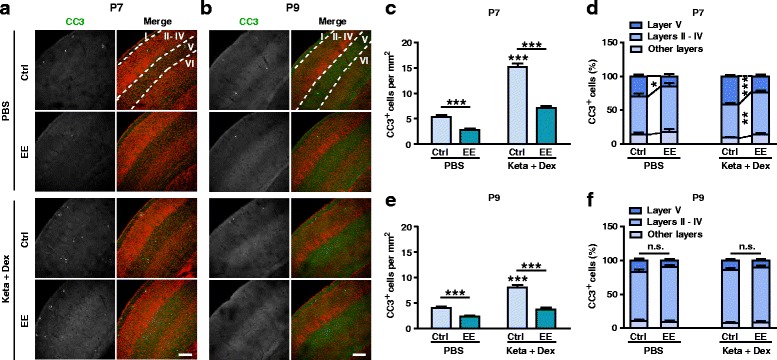
Postnatal EE rearing significantly reduced the extent of apoptosis and promoted a more mature laminar pattern. **a**, **b** Representative confocal images of S1 sections from P7 (**a**) and P9 (**b**) mice labeled with CC3 (green) and TOPRO (red). Treatment conditions as indicated. Cortical layers are indicated by Roman numerals, and borders between layers are marked using dotted lines. Scale bar is 200 μm. **c**, **e** EE rearing significantly reduced the number of CC3^+^ cells in S1 in both PBS and Keta + Dex group at P7 (C) and P9 (E). ^***^ *P* < 0.001, using two-way ANOVA followed by Bonferroni post hoc tests. **d**, **f** Distribution of CC3^+^ cells in different cortical layers at P7 (D) and P9 (F). ^*^ *P* < 0.05, ^**^ *P* < 0.01, ^***^ *P* < 0.001, n.s., not significant, using two-way ANOVA followed by Bonferroni post hoc tests. 4 mice were used per condition at P7, and 4–5 mice were used per condition at P9, 10 brain slices from each mouse were quantitated. Data are shown as the mean ± SEM

The weak effect of hM3Dq on reducing neuronal apoptosis could be because the experiments were carried out at P9, when the apoptosis rate has dropped below its peak level. However, due to the time required for AAV expression, it was not possible to move the time window forward. We thus used an alternative approach, that of rearing mice in an EE. In previous work, we showed that EE rearing from the time of birth (please see Methods section for details) can significantly increase neuronal activity and excitatory synaptic transmission in the cerebral cortex and hippocampus by as early as P7 [27, 29]. Here, using the same paradigm, we found that EE rearing from birth significantly reduced the number of CC3^+^ cells in P7 mice, independent of whether they were injected with PBS (Ctrl: 5.31 ± 0.39 /mm^2^, EE: 2.77 ± 0.26/mm^2^, *P* < 0.001; Fig. 6a, c) or ketamine plus dexmedetomidine (Ctrl: 15.16 ± 0.69/mm^2^, EE: 7.13 ± 0.37/mm^2^, *P* < 0.001; Fig. 6a, c). Furthermore, the laminar pattern of apoptotic cells also shifted towards a more mature phenotypes, with a significant increase in the proportion of layer II – IV cells and a reduction in the proportion of layer V cells (Fig. 6d). When the same experiments were performed in P9 mice, EE rearing reduced the number of CC3^+^ cells in both PBS (Ctrl: 4.03 ± 0.28 /mm^2^, EE: 2.31 ± 0.22/mm^2^, *P* < 0.001; Fig. 6b, e) and ketamine treated (Ctrl: 8.00 ± 0.53/mm^2^, EE: 3.69 ± 0.38/mm^2^, *P* < 0.001; Fig. 6b, e) mice. Similar to the results of the hM3Dq experiment, the laminar pattern of apoptotic cells at P9, already displaying the more mature form, was not significantly affected (Fig. 6f). Together, these results demonstrate that naturally increasing neuronal activity through EE rearing significantly reduced the magnitude of both physiological and ketamine-induced neuronal apoptosis in S1 of P7 and P9 mice, and shifted the distribution of apoptotic cells to the more mature laminar pattern.

**Fig. 7:**
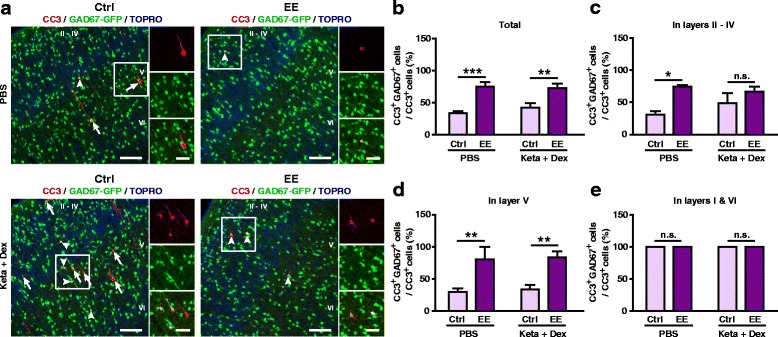
EE rearing altered the proportion of apoptotic GABAergic neurons. **a** Representative confocal images of S1 sections labeled with CC3 (red), GAD67-GFP (green) and TOPRO (blue). CC3^+^ cells co-localizing with GAD67-GFP are indicated by white arrowheads, while those not co-localizing are indicated by white arrows. Conditions as indicated; scale bar is 100 μm. Zoomed image of boxed regions are shown to the right of each panel; scale bar is 50 μm. **b**-**e** Proportion of CC3^+^ cells co-labelling with GAD67-GFP in all cortical layers (**b**), layers II – IV (**c**), layer V (**d**) and layers I and VI (**e**). 3–5 mice were used per condition. ^*^ *P* < 0.05, ^**^ *P* < 0.01, ^***^ *P* < 0.001, n.s., not significant, using two-way ANOVA followed by Bonferroni post hoc tests. Data are shown as the mean ± SEM

Since the cell-type specificity of apoptosis, in addition to its laminar patterning, also changed during development, we next assayed the effect of EE rearing on the proportion of GABAergic CC3^+^ cells using GAD67-GFP transgenic mice reared in EE from birth until P7. We found that EE rearing significantly increased the proportion of cells co-labelling with CC3^+^ and GAD67-GFP^+^ in both PBS (Ctrl: 34.12 ± 2.49%, EE: 75.39 ± 6.45%, *P* < 0.001; Fig. 7a, b) and ketamine plus dexmedetomidine treatment groups (Ctrl: 42.10 ± 7.19%, EE: 73.00 ± 6.62%, *P* < 0.01; Fig. 7a, b). The proportions of apoptotic GAD67^+^ interneurons in layers II – IV and layer V were also increased in EE mice (Fig. 7c, d). In layers I and VI, all apoptotic neurons were GAD67^+^ interneurons in both control and EE reared mice (Fig. 7e). The proportion of apoptotic GAD67-GFP^+^ neurons in P7 EE mice is very similar to that observed in P9 control mice (Fig. 3). Despite the proportion of CC3^+^GAD67^+^ neurons in total CC3^+^ increased by EE rearing, the number of apoptotic GAD67^+^ interneurons did not significantly changed (Ctrl vs EE: PBS, 2.17 ± 0.15/mm^2^ vs 2.52 ± 0.24 /mm^2^, *P* > 0.05; Keta + Dex, 7.63 ± 0.34/mm^2^ vs 6.35 ± 0.32/mm^2^, *P* > 0.05; *N* = 3–5 mice per condition; two-way ANOVA followed by Bonferroni post hoc tests). So, the increase of the proportion of CC3^+^GAD67^+^neurons by EE might be attributed to the reduction of apoptotic pyramidal neurons rather than GAD67^+^ interneurons. Together, these results demonstrate that EE rearing reduced the magnitude of both physiological and ketamine-induced neuronal apoptosis in S1 at P7 and P9 and shift the pattern of apoptosis to a more mature form.

## Discussion

According to the clinical investigations, the adverse effect of general anesthetics on the central nervous system (CNS) is still controversial [44–46]. However, the evidences from animal studies are conclusive, and it depends on the dose of anesthetics, duration or frequency of anesthesia [47, 48]. Although the experimental condition in animal studies cannot totally accord with clinical investigations, the potential hazard of general anesthetics to human should not be ignored. Previous studies have been demonstrated that repeated treatment of ketamine resulted in neurotoxicity/neuroapoptosis in the neonatal CNS [18, 47]. We found that even a single treatment of ketamine could induce cortical apoptosis and it was dose dependent. Moreover, our results indicated that ketamine-induced cortical apoptosis in a dose dependent manner, and it followed a very similar time course to that of physiological apoptosis. Many (approximate 70%) neurons are eliminated during normal brain development, and such physiological apoptosis is critical for the establishment of normal structure of CNS [49]. However, a hypernomic neuronal apoptosis may cause a major disruption in brain development with the potential to permanently shape behavior and cognitive ability [50]. We found that elevating neuronal activity is a possible method for reducing such adverse effect of ketamine.

### Ketamine-induced apoptosis follows a similar pattern to physiological apoptosis

In the current study, we comprehensively analyzed the developmental time course of physiological and ketamine-induced apoptosis in mouse S1 and showed that they followed similar developmental patterns, peaking during P5 – P7, and shifting from primarily layer V pyramidal neurons at P5 to mostly GABAergic interneurons in layers II – IV at P9 (Figs. 1, and 3). By P12, both physiological and ketamine-induced and were not significantly different from each other (Fig. 1).

Previous studies investigating developmental changes in cell numbers in the cerebral cortex reported most significant changes in cortical layers II – IV during P5 – P10 [18, 19] while those examining ketamine-induced apoptosis using CC3 reported high levels of apoptosis in layer II [51]. However, these studies did not assay whether the apoptotic layer II – IV neurons were pyramidal or GABAergic, nor did they quantify whether the number of apoptotic cells in different layers changed during development. In terms of the number of GABAergic cells undergoing apoptosis, previous report showed that under physiological conditions, this process peaked around P7 [20], but did not describe its layer-specific changes during development. Our results add to existing knowledge by showing that physiological and ketamine-induced apoptosis follow similar patterns, as assayed by multiple parameters, including developmental time course, lamination and cell-type specificity. While the similarity does not negate potential adverse effects of anesthesia-induced apoptosis on neural circuit formation and function, they do suggest potentially similar underlying mechanisms.

In terms of dosage, the combination of 30 mg/kg ketamine and 20 μg/kg dexmedetomidine provided an adequate surgical plane of anesthesia, typically for 2–3 h, while maintaining normal oxygenation and respiration (Additional file 2: Table S1). Under these conditions, the number of CC3^+^ cells is about 2.8 fold that of physiological apoptosis at P7. Given that 12.94 ± 0.68 CC3^+^ cells per mm^2^ is less than 1% of cells in the brain (1716.47 ± 37.78/mm^2^ at P7), the long-lasting physiological consequences of one-time, low dose anesthesia are likely to be limited. When the ketamine dose was increased to 60 or 90 mg/kg, more CC3^+^ cells were detected, with the maximum level reached at 60 mg/kg ketamine plus 20 μg/kg dexmedetomidine (21.67 ± 1.66 cells/mm^2^, 4.67 fold compared to control; Fig. 2).

When ketamine was administered alone, paddling limb movements were observed, with higher occurrences at lower doses. To provide an adequate plane of surgery, dexmedetomidine was co-administered at 20 μg/kg. At both 30 and 60 mg/kg ketamine, co-administration with dexmedetomidine increased the number of CC3^+^ cells (Fig. 2). Dexmedetomidine, when administered alone, has no significant effect on apoptosis (Fig. 1). These results differ from previous reports showing that co-administration with dexmedetomidine reduced isoflurane-induced neuronal apoptosis in neonatal rats [52, 53]. One possible explanation for this difference is that ketamine functions by inhibiting NMDA receptors, while isoflurane mainly activates GABA_A_ receptors. Differences in the main mechanisms of action of these drugs may affect how they interact with other agents. Our result suggest that effects of co-administration with dexmedetomidine needs to be examined for individual anesthetic agents. In the case of ketamine, although co-administration with dexmedetomidine increased apoptosis, the extent is still significantly lower than the higher doses of ketamine that would be necessary for maintaining an adequate plane of surgery (Fig. 2).

### Activity-dependence of ketamine-induced apoptosis

In further experiments, we demonstrated that neuronal activity bidirectionally regulated the pattern of ketamine-induced apoptosis (Figs. 4, and 5) and that naturally increasing neuronal activity through EE significantly reduced the level of ketamine-induced apoptosis and shifted its pattern to a more mature form (Figs. 6, and 7). Here, we showed that manipulating the activity of cortical neurons using the DREADD system bidirectionally regulated their susceptibility to ketamine-induced apoptosis, both in terms of CC3^+^ cell number and lamination pattern. When neuronal activity was reduced using AAV-hM4Di-mCitrine, apoptosis rate in both hM4Di-expressing and neighboring non-expressing neurons were increased, suggesting that both cell autonomous and non-cell autonomous mechanisms likely contribute. In contrast to ketamine-induced apoptosis, the DREADD system did not significantly alter the magnitude and lamination pattern of physiological apoptosis (Figs. 4, and 5). However, the activity-dependency of physiological apoptosis has been previously demonstrated using systemic injections of NMDA receptor inhibitors MK-801, PCP and CPP, which significantly increased apoptosis in rodent pups [18, 38], and when naturally increasing neuronal activity through EE, the level of both physiological and ketamine-induced apoptosis were significantly reduced, and the apoptotic patterns were similarly shifted to a more mature form (Figs. 6, and 7). So, we supposed that the DREADD system which locally altered neuronal activity in S1 might not be as strong as EE to impact the level of physiological neuronal apoptosis, but it could alter the threshold of the neurons to ketamine-induced apoptosis.

In all experiments, a shift of the lamination pattern of CC3^+^ cells from mostly layer V pyramidal neurons at P5 (or lowered activity) to mostly layers II – IV GABAergic neurons (or elevated activity) could be observed. What physiological mechanism or process could underlie this developmental change? The cortical plate is formed by migrated neurons in an inside-out manner [53–56], with earlier generated neurons occupying the deeper layers of the cortex and later generated neurons migrating beyond them to form the upper layers. Therefore, neurons in the upper layers are generally less mature than those in the deeper layers of the cerebral cortex. The apoptosis pattern we observed is consistent with developmental age, in that deep layer neurons are more vulnerable at earlier ages.

The more intriguing and harder-to-explain shift is that from mostly glutamatergic pyramidal neurons at P5 to mostly GABAergic neurons at P9. Since GABAergic neurons need to travel longer distances and undergo both tangential and radial migration to reach the cerebral cortex [53–57], the exact age correspondence between pyramidal and GABAergic neurons in each cortical layer is difficult to determine. In general terms, however, early-born medial ganglionic eminence (MGE) cells mostly populate deep layers (V – VI), while later born MGE cells mostly populate superficial layers (II – IV) [58], consistent with the inside-out development of the cerebral cortex. Recent studies suggest that deep and superficial layer neurons may derive from distinct groups of progenitor cells, both for pyramidal and GABAergic neurons [58]. According to this hypothesis, it is possible that deep layer pyramidal neurons and superficial GABAergic neurons may be more vulnerable to apoptosis, through intrinsically determined developmental mechanisms. Recent experiments showed that transplanted GABAergic neurons underwent apoptosis according to their own developmental age, rather than the age of the recipient cortex [20, 59, 60]. Furthermore, the apoptosis rate of neither transplanted nor endogenous GABAergic neurons varied with the number of transplanted cells, suggesting that intrinsic developmental programs, rather than extrinsic competition-based mechanisms, regulated apoptosis of GABAergic neurons [20, 59]. We surmise that the same intrinsic programs may also regulate the apoptosis of pyramidal neurons, and ketamine may augment the normal developmental programing by lowering the threshold of the intrinsic apoptotic program.

### Enrichment protected neurons from ketamine-induced apoptosis

An important result of our study is that EE rearing from birth significantly reduced the level of both physiological and ketamine-induced apoptosis and shifted the apoptotic pattern to a more mature one (Figs. 5, and 6). EE paradigms use a combination of complex in animate and social stimulations to promote brain development and plasticity in rodents [61]. Previous studies have shown that EE rearing can elicit structural and functional changes in the nervous system, as well as rescue functional deficits in various neurological disorders [61–64]. In previous work, we showed that EE rearing from birth can promote glutamatergic and GABAergic synaptic transmission, as well as increase cortical and hippocampal levels of brain-derived neurotrophic factor (BDNF), as early as P7 [27, 29]. These molecular and cellular changes could provide mechanistic bases for the observed maturation in lamination pattern of physiological and ketamine-induced apoptosis.

Since surgery and anesthesia are sometime unavoidable in young children, our results suggest that increased natural sensory stimulation through EE before and after surgery may significantly reduce the adverse effects of anesthesia on increasing apoptosis. Based on our understanding of the effects of EE, its beneficial effects likely also extend to other aspects of brain development and function.

## Additional files

Additional file 1: Figure S1. Expression of AAV in S1 was not significantly different in each group. **(A)** Proportion of Nissl^+^ cells that are hM4Di-mCitrine^+^, conditions as indicated. n.s., not significant, using two-way ANOVA followed by Bonferroni post hoc test. 3 mice were used per condition. **(B)** Proportion of Nissl^+^ cells that are hM3Dq-mCitrine^+^, conditions as indicated. n.s., not significant, using two-way ANOVA followed by Bonferroni post hoc test. 4 mice were used per condition. Data are shown as the mean ± SEM. (PDF 72 kb)

Additional file 2: Table S1. Arterial blood gas analysis of P7 mice. (DOCX 16 kb)

## Abbreviations

BDNF: Brain-derived neurotrophic factor
BSA: Bovine serum albumin
CC3: Cleaved caspase-3
CNO: Clozapine-N-oxide
CNS: Central nervous system
CPP: Carboxypiperazin-4-yl-propyl-1-phosphonic acid
Dex: dexmedetomidine
DREADDs: Designer receptors exclusively activated by designer drugs
E: Embryonic days
EE: Environmental enrichment
Keta: ketamine
MGE: Medial ganglionic eminence
NMDA: N-Methyl-D-aspartic acid
P: Postnatal ages
PaCO_2_: Partial pressure of carbon dioxide
PaO_2_: Pressure of oxygen
PBS: Phosphate-buffered saline
PCP: Phencyclidine
PFA: Paraformaldehyde
S1: Somatosensory cortex
SaO_2_: Oxygen saturation
