# Supplementary figures and images for: Ketamine-induced apoptosis in the mouse cerebral cortex follows similar characteristic of physiological apoptosis and can be regulated by neuronal activity
Source: Mol Brain. 2017 Jun 17;10:24. doi: 10.1186/s13041-017-0302-2 (PMC5474024; doi:10.1186/s13041-017-0302-2)

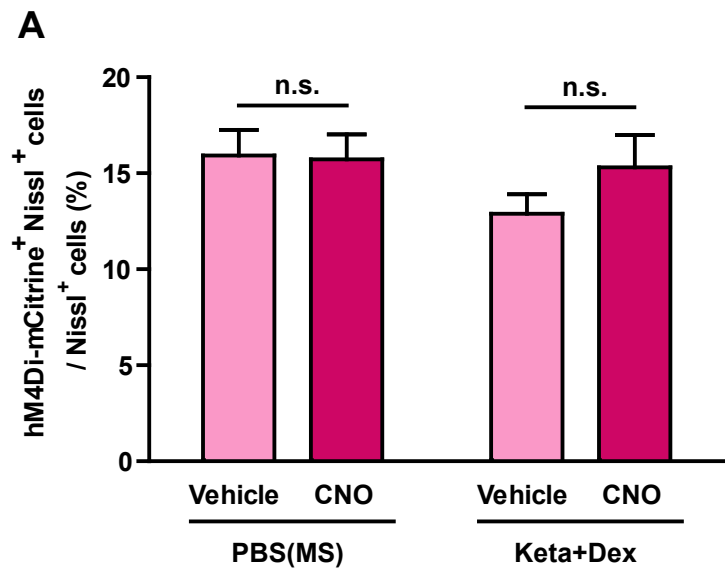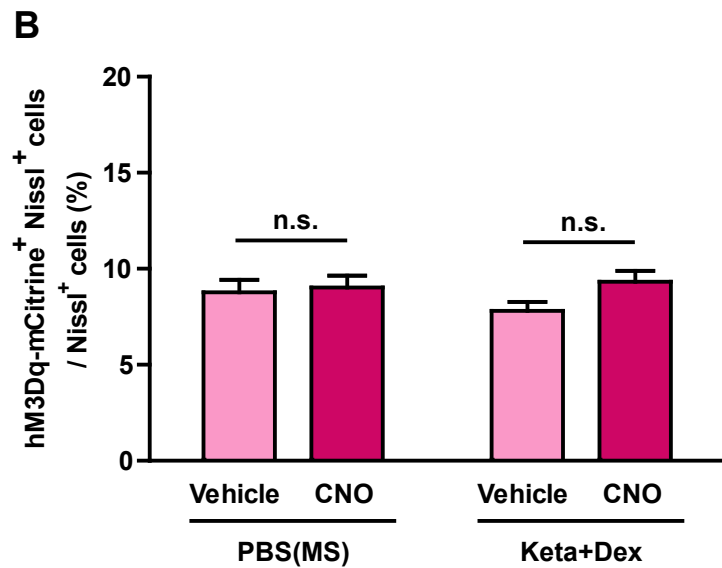

Supplementary Figure 1, Wang et al.

Supplement: Supplementary file 1 — Expression of AAV in S1 was not significantly different in each group. (A) Proportion of Nissl+ cells that are hM4Di-mCitrine+, conditions as indicated. n.s., not significant, using two-way ANOVA followed by Bonferroni post hoc test. 3 mice were used per condition. (B) Proportion of Nissl+ cells that are hM3Dq-mCitrine+, conditions as indicated. n.s., not significant, using two-way ANOVA followed by Bonferroni post hoc test. 4 mice were used per condition. Data are shown as the mean ± SEM. (PDF 72 kb) [file 13041_2017_302_MOESM1_ESM.pdf]
