# Supplementary material for: Ketamine-induced apoptosis in the mouse cerebral cortex follows similar characteristic of physiological apoptosis and can be regulated by neuronal activity
Source: Mol Brain. 2017 Jun 17;10:24. doi: 10.1186/s13041-017-0302-2 (PMC5474024; doi:10.1186/s13041-017-0302-2)
Supplement: Supplementary file 2 — Arterial blood gas analysis of P7 mice. (DOCX 16 kb) [file 13041_2017_302_MOESM2_ESM.docx]

**Supplementary Table. Arterial blood gas analysis of P7 mice.**

| **Parameter** | **Ctrl** | **Keta 30mg/kg** | | **Keta 30mg/kg+**  **Dex 20μg/kg** | | **Keta 60mg/kg** | | **Keta 60mg/kg+**  **Dex 20μg/kg** | | **Keta 90mg/kg** | | **Keta 90mg/kg+**  **Dex 20μg/kg** | |
| --- | --- | --- | --- | --- | --- | --- | --- | --- | --- | --- | --- | --- | --- |
|  | **—** | **0.5 h** | **2.5 h** | **0.5 h** | **2.5 h** | **0.5 h** | **2.5 h** | **0.5 h** | **2.5 h** | **0.5 h** | **2.5 h** | **0.5 h** | **2.5 h** |
| **pH** | 7.55  ± 0.03 | 7.54  ± 0.07 | 7.47  ± 0.01 | 7.46  ± 0.04 | 7.41  ± 0.04 | 7.50  ± 0.05 | 7.44  ± 0.04 | 7.39  ± 0.04 | 7.34  ± 0.06 | 7.46  ± 0.06 | 7.41  ± 0.05 | 7.37  ± 0.05 | 7.33*****  ± 0.02 |
| **PaCO_2_**  **(mmHg)** | 26.73  ± 2.15 | 27.48  ± 6.20 | 31.64  ± 1.43 | 27.76  ± 3.69 | 37.82  ± 4.75 | 29.33  ± 3.96 | 37.60  ± 5.43 | 35.36  ± 3.53 | 43.28  ± 8.37 | 32.66  ± 4.49 | 40.12  ± 6.13 | 40.27  ± 6.66 | 44.73  ± 4.63 |
| **PaO_2_**  **(mmHg)** | 107.33  ± 12.92 | 106.00  ± 18.58 | 97.80  ± 2.67 | 102.00  ± 13.28 | 131.40  ± 13.05 | 104.00  ± 13.61 | 96.25  ± 18.02 | 112.56  ± 13.15 | 115.75  ± 18.18 | 106.20  ± 18.54 | 95.40  ± 22.87 | 84.71  ± 16.94 | 76.50  ± 16.22 |
| **HCO_3_^-^**  **(mM)** | 22.90  ± 0.94 | 21.36  ± 2.68 | 22.50  ± 0.53 | 18.84  ± 1.40 | 22.56  ± 1.30 | 20.40  ± 1.04 | 24.35  ± 1.24 | 20.36  ± 0.78 | 21.73  ± 1.86 | 22.52  ± 2.65 | 23.74  ± 0.95 | 21.01  ± 1.67 | 22.37  ± 1.62 |
| **SaO_2_**  **(%)** | 98.35  ± 0.45 | 96.70  ± 1.65 | 98.06  ± 0.22 | 96.87  ± 1.02 | 98.58  ± 0.63 | 95.47  ± 1.46 | 96.38  ± 1.16 | 95.47  ± 2.20 | 96.73  ± 1.59 | 95.94  ± 2.07 | 90.46  ± 5.73 | 89.84  ± 2.99 | 86.88*****  ± 5.00 |

Parameters measured and anesthesia conditions as indicated. Measurements were taken at 0.5 h or 2.5 h after anesthesia. 4 – 10 mice were used per condition. **^*^***P* < 0.05, one-way ANOVA followed by Dunnett’s multiple comparisons tests.
